# AhR-Induced Anti-Inflammatory Effects on a Caco-2/THP-1 Co-Culture Model of Intestinal Inflammation Are Mediated by PPARγ

**DOI:** 10.3390/ijms252313072

**Published:** 2024-12-05

**Authors:** Gustavo Henrique Oliveira da Rocha, Claudia Müller, Susanne Przybylski-Wartner, Heidrun Schaller, Sina Riemschneider, Jörg Lehmann

**Affiliations:** 1 Department of Preclinical Development and Validation, Fraunhofer Institute for Cell Therapy and Immunology, 04103 Leipzig, Germany; gustavo.henrique.oliveira.da.rocha@izi-extern.fraunhofer.de (G.H.O.d.R.); claudia.mueller@izi.fraunhofer.de (C.M.); susanne.przybylski-wartner@medizin.uni-leipzig.de (S.P.-W.); heidrun.schaller@gmx.de (H.S.); sina.riemschneider@izi.fraunhofer.de (S.R.); 2 Fraunhofer Cluster of Excellence Immune-Mediated Diseases [CIMD], 04103 Leipzig, Germany

**Keywords:** aryl hydrocarbon receptor, peroxisome proliferator-activated receptor gamma, inflammatory bowel disease

## Abstract

The aryl hydrocarbon receptor (AhR) and the peroxisome proliferator-activated receptor γ (PPARγ) are ligand-activated transcription factors that have in recent years been investigated for their anti-inflammatory properties for treatment of inflammatory bowel diseases (IBDs). These are globally prevalent chronic maladies of the gut that lack cost-efficient therapeutical options capable of inducing long-term remission. In the present study, we used an in vitro Transwell^®^ co-culture model composed of Caco-2 epithelial cells in the apical compartment and lipopolysaccharide-treated (LPS) THP-1 macrophages in the basolateral compartment. Secretion of cytokines, disruption of epithelial integrity, and expression of surface markers and junctional proteins were assessed in order to investigate interactions between AhR and PPARγ on the ligand-elicited effects on the control of inflammation. The results revealed that the potent AhR ligand 6-formylindolo[3,2-b]carbazole (FICZ) attenuated LPS-induced IL-6 release by macrophages, which then stabilized Caco-2 monolayer permeability by decreasing claudin-2 expression. These effects were disrupted by GW9662 and to some extent by CH223191, inhibitors of PPARγ and AhR, respectively. Our main findings evidence PPARγ might be a downstream regulator of AhR activation essential for its ligand-based anti-inflammatory effects, suggesting it might be employed as either an auxiliary target or as a biomarker of therapeutical efficacy on AhR-based IBD pharmacotherapy.

## 1. Introduction

The aryl hydrocarbon receptor (AhR) is a ligand-activated transcription factor traditionally associated with toxic responses elicited by xenobiotics, especially dioxin-like molecules such as 2,3,7,8-tetrachlorodibenzo-p-dioxin [1]. Toxic effects of AhR-binding xenobiotics are exerted through the translocation of activated AhR into the nucleus after coupling to the AhR nuclear transporter (ARNT) protein, which binds to the xenobiotic response element (XRE) in promoter regions of AhR-activated specific genes, e.g., enzymes of the CYP450 family *CYP1A1*, *CYP1A2*, and *CYP1B1* [2]. While traditional research on AhR has for long been focused mostly on investigating the deleterious effects of its activation by xenobiotics [3,4], in recent years, research on AhR has shifted its focus to addressing newly discovered anti-inflammatory and immunomodulatory effects of naturally occurring exogenous ligands found in diet and/or microbiota, as well as endogenous ligands [1,5].

The compound known as 6-formylindolo[3,2-b]carbazole (FICZ) is among the most potent endogenous AhR ligands, displaying anti-inflammatory actions and being frequently investigated as a promising candidate for treatment of several human conditions, ranging from kidney injury to skin diseases [6,7]. It is formed as a derivative of tryptophan metabolism with the aid of UV light and is quickly degraded by CYP1-metabolizing enzymes following a negative feedback loop signaling mechanism [8]. Most immunomodulating effects of FICZ and other AhR ligands, however, are not exerted through well-established canonical AhR activation pathways, which can lead to deleterious toxic effects, especially at high concentrations, due to generation of reactive oxygen species by CYP1 enzymes [2]. Some of these non-canonical pathways include, for example, activation of estrogen receptors preventing proliferation of breast cancer cells [9] and control of Th17/Treg populations via STAT3 [10]. These non-canonical effects can be elicited by a plethora of endogenous and exogenous ligands, making AhR quite a promiscuous receptor, and it follows that not all of the mechanisms involving AhR activation leading to beneficial immune-mediating effects have been elucidated [11]. Similarly, downstream mediators of non-canonical AhR activation by immunomodulatory ligands such as FICZ are also still largely unknown and not yet fully understood.

One such factor that might play a role in controlling non-canonical AhR-mediated effects is the peroxisome proliferator-activated receptor gamma (PPARγ). This receptor was long associated mostly with control of lipid metabolism, its ligands having been successfully employed on pharmacotherapy of type II diabetes for over two decades [12]. In the past few years, much like AhR, research on PPARγ has diverted to the exploration of its anti-inflammatory effects, especially aiming at improving the pharmacotherapy of inflammation-based diseases that so far lack proper treatment options, which range from neurodegenerative to autoimmune disorders [13,14]. Traditional PPARγ agonists, which refer mostly to thiazolidinediones, have been shown to be effective in the control of inflammation through varied mechanisms in different in vitro and in vivo models, such as decreasing generation of reactive oxygen species, preventing the degradation of the extracellular matrix by inhibiting metalloproteinases, reducing secretion of inflammatory cytokines such as TNF-α and MCP-1, and regulating STAT3 and NF-kB signaling pathways [15,16]. PPARγ itself has also been evidenced as a mediator of the actions of newly discovered anti-inflammatory compounds of natural origin, either due to them directly binding to PPARγ or due to promoting PPARγ-elicited downstream effects [17]. PPARγ has also been shown as a modulator of the effects of other endogenous molecules of known anti-inflammatory actions [18,19]. Indeed, recent research has cemented the central role of PPARγ in controlling inflammation in several different cell types and biological systems.

Inflammatory bowel diseases (IBDs), comprising ulcerative colitis (UC) and Crohn’s disease (CD), refer to a chronic debilitating condition characterized by extensive inflammatory damage affecting the epithelial lining of the gut, which afflicts millions worldwide [20]. Other forms of IBDs such as microscopic colitis and indeterminate IBDs also exist, but these are neither as serious nor as prevalent as UC and CD [21,22]. IBDs develop across a long time period, with their onset hard to pinpoint and dependent on individual genetic factors in combination with continuous exposure to factors mostly associated with a “Western lifestyle”, such as a fat-rich diet and indiscriminate use of non-steroidal anti-inflammatory drugs and antibiotics [23,24,25]. These constitute cumulative microaggressions that eventually lead to damage of the epithelial lining of the gut, triggering microorganism translocation responsible for activating local leukocytes, which in turn release inflammatory mediators, further damaging the gut epithelium in an uncontrolled manner [26]. Among these leukocytes, macrophages are especially important, as they have been found to be key drivers of inflammation during the development of IBD, releasing pro-inflammatory cytokines such as TNF-α, IL-6, and IL-1β in response to invading commensal bacteria and being a major component of inflammatory granulomas in CD. Clinical remission induced by biological therapy is also associated with reduced activity of inflammatory M1 macrophages and a diminished recruitment of peripheral monocytes to the gut [27,28].

While available pharmacotherapy for treatment of IBDs is somewhat effective in achieving clinical remission in most patients, it routinely fails after long term use and is not effective for a substantial number of patients. Mainline therapy options, such as biologicals, are also prohibitively expensive, making the development of new drugs or repurposing of existing ones a necessity in order for these diseases to ever be successfully treated [29]. In this context, endogenous AhR ligands such as FICZ might be promising alternatives for treatment of IBDs, as their beneficial effects have been evidenced in several preclinical studies employing in vivo colitis models [30,31]. To date, however, while AhR agonists such as Tapinarof (Benvitimod) have successfully gone through clinical trials for treatment of epidermal inflammation [32], no AhR ligand, neither natural nor synthetic, has reached clinical trial stages for treatment of IBDs. This is due in part to the complex and not yet fully understood pathogenesis of IBDs and also to the not entirely discovered roles of AhR activation in regard to control of inflammation vs. promotion of tumorigenicity by certain xenobiotics. Thus, the myriad of pleiotropic effects involved with AhR activation is also an aggravating factor [33]. In this context, PPARγ, which by itself has also been identified as playing an important role in controlling inflammation during IBDs by decreasing the activity of inflammatory macrophages [34,35,36], might act as a mediator of downstream effects of AhR activation. Therefore, identifying crucial interactions between both factors might help elucidating the complex mechanisms associated with AhR activation in the inflamed gut, representing a step forward in bringing AhR ligands closer to clinical application.

In order to address these research questions, several in vitro and in vivo models for the study of IBDs are available. Among in vivo models, those employing chemicals such as dextran sulfate sodium (DSS) and 2,4,6-trinitrobenzene sulfonic acid (TNBS) to damage the epithelial lining and induce a colitis-like syndrome in mice are the most common [37,38]. Patient-derived 3D organoids can also be generated from epithelial stem cells obtained from gut biopsies and built as a “gut-like” system that better represents actual disease and is very useful in the context of therapy individualization and intestinal disease modeling [39,40]. While these models are useful for efficacy studies, the cumbersomeness in standardization, the complexity of disease onset events, and the difficulty in pinpointing specific time points involving leukocyte activation make it challenging to use animal models and organoids for mechanistic studies, especially when trying to determine the role of immune cells [41,42]. On the other hand, in vitro models utilizing the colonic human cell line Caco-2 alongside the human monocyte leukemia cell line THP-1 in a Transwell^®^ co-culture system represent a well-established “leaky gut” model for mode of action studies of potential IBD therapeutics. Not only does the model consist of cells of human origin, it also permits a tighter control of the inflammatory response elicited by macrophage-differentiated THP-1 cells, allowing for a more focused evaluation of endpoints that better address local inflammation [43,44].

The present study aimed to investigate whether there is a pivotal crosstalk between AhR and PPARγ potentially contributing to the anti-inflammatory and barrier-homeostasis restoring effects of the highly efficient endogenous AhR agonist FICZ. After initial screening on an open RNA-seq database derived from gut macrophages of UC patients, a “leaky gut” co-culture model utilizing Caco-2 and macrophage-differentiated THP-1 cells robustly mimicking IBD in vitro was employed. Significant effects elicited by PPARγ and AhR alone or synergistically mediated by both receptors were identified.

## 2. Results

### 2.1. PPARG and AHR Transcriptional Levels Correlated in Gut Macrophages of Healthy Individuals, but Not in Macrophages of UC Patients

**Figure 1:**
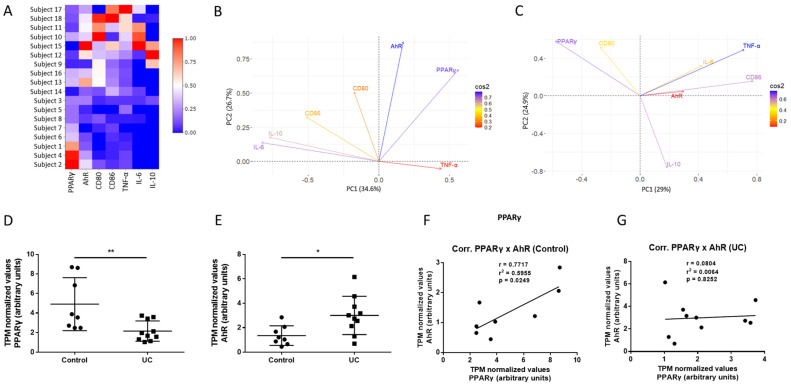
**Associations between gene expression levels of *PPARG*, *AHR*, and macrophage inflammatory markers in a GEO IBD dataset.** RNA-seq data from an IBD dataset deposited at GEO was screened for transcriptional activation of *PPARG*, *AHR*, *CD80*, *CD86*, *TNFA*, *IL6*, and *IL10* genes among healthy controls and UC patients. After being normalized to a 0–1 scale (range represented in the scalebar), values were displayed in a heatmap following a numerical order of subjects (**A**). PCA analyses were carried out individually for both the healthy control and UC groups. PC1 and PC2 represent principal components 1 and 2, respectively. The scale bar represents cos2 values, which indicate the color of the eigenvectors denoting how well they are represented by the variance model (**B**,**C**). TPM values (transcripts per million) for each of the parameters assessed were individually plotted, averaged, and compared between healthy controls and UC patients. Results are shown as mean ± standard deviation of the mean and were analyzed via Student’s *t* test. * *p* ≤ 0.05, ** *p* ≤ 0.01 in comparison between groups (**D**,**E**). Correlation tests between *AHR* and *PPARG* utilizing TPM values for both healthy control and UC patient groups were performed and individual positions of every correlation pair alongside the exact value for Pearson’s r correlation coefficient, *r*^2^, and *p* value, are shown (**F**,**G**). For all analyses, a total *n* = 8 for healthy controls and *n* = 10 for UC patients were utilized.

In order to better support our starting hypothesis, we initially accessed RNA-seq data from a Gene Expression Omnibus (GEO) dataset generated from gut macrophages of UC patients and healthy controls and tried to determine whether there could be any changes or associations between *AHR* and *PPARG* transcriptional levels alongside common macrophage markers in IBDs, such as inflammatory cytokines *TNFA*, *IL6*, and *IL10* and surface markers *CD80* and *CD86*. Initial observations demonstrated a clear difference in grouping of the study subjects based on whether they were healthy controls or UC patients, especially pertaining to *PPARG* gene expression levels in comparison to the levels of other assessed markers (Figure 1A). Next, principal component analyses (PCA) revealed that in the healthy control group, both *AHR* and *PPARG* expression contribute the most to the main principal component while their respective vectors also point in a same direction, suggesting there is a likely correlation between both factors (Figure 1B). In contrast, PCA tests carried out for the UC patient group evidenced that other inflammatory factors become more relevant in explaining variance of the model, as *TNFA* becomes the most relevant vector for the main principal component in at the expense of *AHR*. *PPARG*, however, remains relevant to the model, suggesting it might act as a regulator during UC. Also, vectors for *PPARG* and *AHR* point in opposite directions in the UC patient group, meaning any correlations between *PPARG* and *AHR* are lost during disease (Figure 1C).

Indeed, direct comparison between healthy control and UC patient groups showed that *PPARG* expression levels were significantly higher in the healthy control group in comparison to the UC patient group, while *AHR* expression levels were found to be significantly lower in the healthy control group in comparison to the UC patient group (Figure 1D,E), with the same being true also for all other markers assessed (Supplementary Figure S1A–E), showing that both *PPARG* and *AHR* gene expression levels changed during UC progression. Further Pearson correlation analyses confirmed *PPARG* and *AHR* to be positively and significantly correlated in healthy controls, but no longer in UC patients (Figure 1F,G). This suggests there is a fine regulated control between *AHR* and *PPARG* levels in gut macrophages to sustain homeostatic signaling. Thus, alterations in the expression levels of both *AHR* and *PPARG* during the course of UC might compromise this regulatory system.

### 2.2. FICZ Treatment Decreased PPARγ Protein Expression

**Figure 2:**
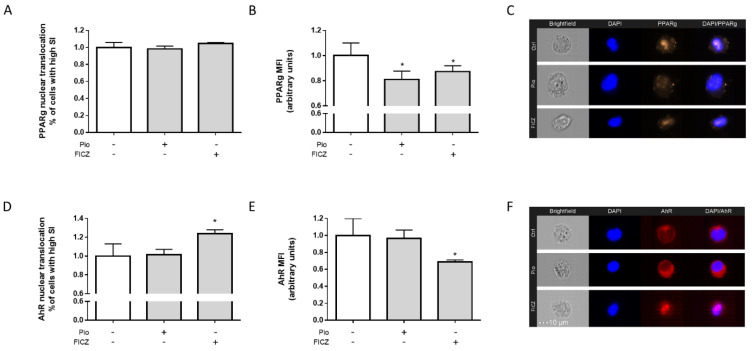
**Effects of agonists on nuclear translocation and protein expression of AhR and PPARγ in THP-1 cells.** Macrophage-differentiated THP-1 cells were treated with 10 µM of each pioglitazone (Pio) and FICZ for 24 h. Cells were harvested, fixed, and stained after treatments. Nuclear translocation (**A**,**D**) and overall protein expression (**B**,**E**) of PPARγ and AhR, respectively, were assessed via imaging flow cytometry; representative images of the treatment groups are also shown (**C**,**F**). Data are displayed as normalized % of cells of high similarity indexes for translocation and as normalized MFI values for expression. Results are shown as mean ± standard deviation of the mean (*n* = 4). Statistical significance was verified by one-way ANOVA followed by Tukey’s post hoc test. * *p* ≤ 0.05 in comparison to the untreated control group. “+” indicates the respective treatment at the left side of the graph was carried out; “-” indicates it was not.

After having established a correlation between *PPARG* and *AHR* levels in human macrophages based on RNA-seq data, we next aimed to determine the direct effects of pioglitazone (selective PPARγ agonist) and FICZ (potent AhR agonist) on receptor activation and expression in THP-1 macrophages in order to further assess the extent of such correlation. No effects were seen on PPARγ translocation, regardless of the agonist used (Figure 2A), but its expression was reduced under treatment with pioglitazone. Notably, FICZ also induced a reduction in PPARγ expression (Figure 2B). These effects on both expression and translocation of PPARγ are both visually noticeable on imaging flow cytometry images (Figure 2C). As for AhR, treatment with FICZ induced its nuclear translocation, as expected (Figure 2D). Analogous to the effects observed for PPARγ, FICZ decreased overall expression of AhR (Figure 2E). These effects can be visually assessed on imaging flow cytometry images (Figure 2F). PPARγ expression being reduced by pioglitazone points to a natural negative feedback response within macrophages to PPARγ agonism, and the same is true for AhR expression being reduced by FICZ. The fact that FICZ treatment also reduced PPARγ expression while inducing AhR translocation indicates it might recruit PPARγ downstream of AhR translocation, generating a negative feedback response affecting PPARγ.

### 2.3. AhR Nuclear Translocation Was Blocked by PPARγ Inhibition in THP-1 Macrophages

**Figure 3:**
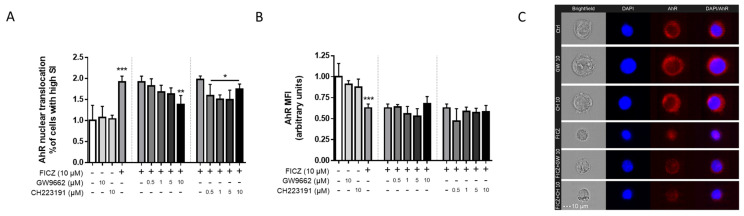
**Effects of inhibitors on FICZ-mediated nuclear translocation and protein expression of AhR in THP-1 cells.** Macrophage-differentiated THP-1 cells were treated with 10 µM FICZ, 10 µM GW9662 (GW), and 10 µM CH223191 (CH), either alone or combined, for 24 h. Cells were harvested, fixed, and stained after treatments. Nuclear translocation (**A**) and overall expression (**B**) of AhR were assessed via imaging flow cytometry; representative images of the treatment groups are also shown (**C**). Data are displayed as normalized % of cells of high similarity indexes for translocation and as normalized MFI values for expression. Results are shown as mean ± standard deviation of the mean (*n* = 4). Statistical significance was verified by one-way ANOVA followed by Tukey’s post hoc test. * *p* ≤ 0.05, ** *p* ≤ 0.01, *** *p* ≤ 0.001 in comparison to the untreated control group (first dataset) or LPS-treated group (second and third datasets). “+” indicates the respective treatment at the left side of the graph was carried out; “-” indicates it was not. Dotted vertical lines separate datasets. The small horizontal line groups columns together.

Having established that PPARγ expression in THP-1 macrophages is affected by FICZ treatment, we next assessed whether PPARγ is also important for FICZ-mediated AhR nuclear translocation. Treatment with different concentrations of GW9662 (selective PPARγ inhibitor) prevented AhR translocation induced by FICZ in a dose-dependent manner, with the effect being significant at the highest concentration. This shows that PPARγ activity is crucial for proper translocation of AhR into the nucleus after its activation by an agonist such as FICZ. We also ran a similar experiment utilizing different concentrations of CH223191 (potent AhR inhibitor) to control for optimal inhibitor concentrations to be used in the next experiments, given that CH223191 can be displaced by ligands such as FICZ. The results showed that under the conditions tested, CH223191 is an effective inhibitor of FICZ-induced AhR translocation, as even at low concentrations, the inhibitory effect was observed (Figure 3A). Of note, the previously seen effect of FICZ reducing AhR expression was not abrogated by any inhibitors, neither GW9662 nor CH213191, suggesting that an indirect downstream effect elicited by the presence of FICZ rather than direct activation of AhR is responsible for the downregulation of AhR expression (Figure 3B). These effects can be visually assessed on imaging flow cytometry images (Figure 3C).

### 2.4. Expression of Inflammatory Surface Markers Was Modulated by AhR and PPARγ Independently of One Another in LPS-Treated THP-1 Macrophages

**Figure 4:**
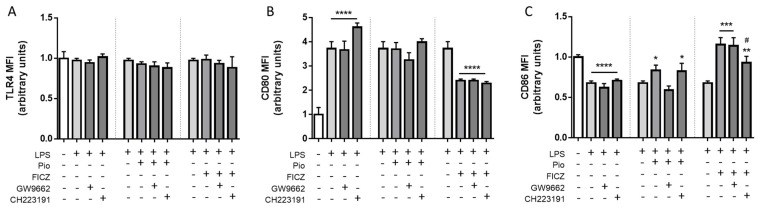
**Expression of inflammatory surface markers in THP-1 cells.** Macrophage-differentiated THP-1 cells were treated with 100 ng/mL LPS alongside 10 µM pioglitazone (Pio), 10 µM FICZ, 10 µM GW9662, and 10 µM CH223191, either alone or combined, for 24 h. Cells were harvested and stained after treatments. Expression of surface markers TLR4 (**A**), CD80 (**B**), and CD86 (**C**) were assessed by conventional flow cytometry. Data are displayed as normalized MFI values. Results are shown as mean ± standard deviation of the mean (*n* = 4). Statistical significance was verified by one-way ANOVA followed by Tukey’s post hoc test. * *p* ≤ 0.05, ** *p* ≤ 0.01, *** *p* ≤ 0.001, **** *p* ≤ 0.0001 in comparison to the untreated control group (first dataset) or the LPS-treated group (second and third datasets). # *p* ≤ 0.05 in comparison to the LPS-treated group (first dataset) or the Pio/FICZ-treated group (second and third datasets). “+” indicates the respective treatment at the left side of the graph was carried out; “-” indicates it was not. Dotted vertical lines separate datasets. Small horizontal lines group columns together.

Having determined how PPARγ and AhR respond to the ligands used in terms of expression and translocation, we next aimed to investigate whether any other potential relationship between both factors would exert any influences on the anti-inflammatory actions displayed by both. To this end, we assessed the effects of ligands on lipopolysaccharide (LPS)-treated THP-1 macrophages, starting with surface markers. We initially determined that none of the ligands had any inhibitory effects on toll-like receptor 4 (TLR4) surface expression, meaning any anti-inflammatory effects caused by PPARγ and AhR agonists would be attributed to downstream targeting rather than reduced binding of LPS to TLR4 (Figure 4A). After 24 h of LPS treatment, we verified an increase in CD80 and decrease in CD86 levels, a polarization profile associated with a prolonged M2b response prevalent at UC onset. Under these conditions, pioglitazone was able to reverse the decrease in CD86 caused by LPS (effect prevented by GW9662), while FICZ reversed the effects caused by LPS on both CD80 and CD86 (effect on CD86 diminished by CH223191). However, AhR inhibition did not prevent the effects of pioglitazone and neither did PPARγ inhibition prevent the effects of FICZ, suggesting that FICZ is still capable of modulating expression of IBD-linked inflammatory surface markers independently of AhR translocation into the nucleus (Figure 4B,C).

### 2.5. Secretion of IL-6 Was Modulated Simultaneously by Both AhR and PPARγ in LPS-Treated THP-1 Macrophages

**Figure 5:**
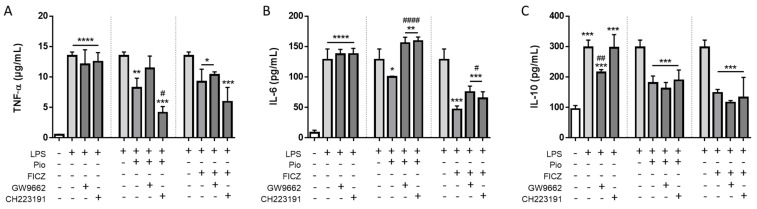
**Secretion of proinflammatory cytokines by THP-1 cells.** Macrophage-differentiated THP-1 cells were treated with 100 ng/mL LPS alongside 10 µM pioglitazone (Pio), 10 µM FICZ, 10 µM GW9662, and 10 µM CH223191, either alone or combined, for 24 h. Supernatants were collected after treatments. Secretion of cytokines TNF-α (**A**), IL-6 (**B**), and IL-10 (**C**) were assessed by ELISA. Data are displayed as [µg/mL] for TNF-α and [pg/mL] for IL-6 and IL-10. Results are shown as mean ± standard deviation of the mean (*n* = 4). Statistical significance was verified by one-way ANOVA followed by Tukey’s post hoc test. * *p* ≤ 0.05, ** *p* ≤ 0.01, *** *p* ≤ 0.001, **** *p* ≤ 0.0001 in comparison to the untreated control group (first dataset) or the LPS-treated group (second and third datasets). # *p* ≤ 0.05, ## *p* ≤ 0.01, #### *p* ≤ 0.0001 in comparison to the LPS-treated group (first dataset) or the Pio/FICZ-treated group (second and third datasets). “+” indicates the respective treatment at the left side of the graph was carried out; “-” indicates it was not. Dotted vertical lines separate datasets. Small horizontal lines group columns together.

After establishing that there is no direct relationship between PPARγ and AhR regarding their effects on the expression of inflammatory surface markers, we then assessed whether effects elicited by either FICZ or pioglitazone on cytokine secretion could influence each other. Indeed, both ligands were able to reverse the increased secretion of all inflammation-related cytokines assessed in the supernatant from the basolateral compartment of the co-culture model, namely, TNF-α, IL-6, and IL-10. These cytokines are characteristic of the M2b polarization profile; are highly secreted during the course of IBD; and in the case of TNF-α and IL-6, disrupt epithelial barrier integrity. Treatment with GW9662 inhibited the decrease in cytokine secretion elicited by pioglitazone, but curiously, CH223191 was not effective in preventing similar effects elicited by FICZ for TNF-α and IL-10, once again demonstrating that FICZ might be capable of displaying anti-inflammatory effects that are not only independent of AhR translocation but also partially or completely independent of binding to AhR. Still, FICZ-mediated prevention of LPS-induced cytokine secretion was abrogated by CH223191 for IL-6, indicating this cytokine is modulated by an AhR-mediated mechanism. Also, reduction of IL-6 secretion was prevented by the inhibitors in a reciprocal manner: GW9662 inhibited FICZ-mediated IL-6 reduction, while CH223191 inhibited pioglitazone-mediated reduction. These results show that IL-6 is a central inflammatory cytokine whose secretion is mediated simultaneously by both PPARγ and AhR activation, likely under a common pathway. Of note, cytokine levels measured in the apical compartment were below detection limits, meaning the cytokines detected in the basolateral compartment were mostly originated from the THP-1 macrophages and are thus the ones represented in the graphs. (Figure 5A–C).

### 2.6. Epithelial Permeability and Protein Expression of Claudin-2 in the LPS-Treated Caco-2/THP-1 Co-Culture Model Was Modulated by FICZ in a PPARγ-Dependent Manner

**Figure 6:**
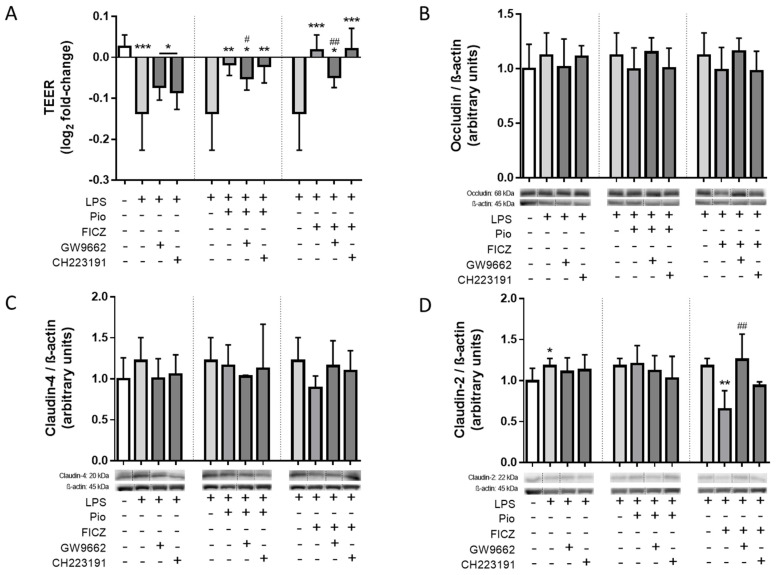
**Epithelial integrity and expression of tight junction proteins in Caco-2 cells.** Caco-2 cells were placed in a co-culture system alongside macrophage-differentiated THP-1 cells and were treated with 100 ng/mL LPS alongside 10 µM pioglitazone (Pio), 10 µM FICZ, 10 µM GW9662, and 10 µM CH223191, either alone or combined, for 24 h. TEER measurements on the Caco-2 monolayer were performed immediately before and after the treatment period (**A**). Caco-2 cells were also harvested in RIPA buffer, and the expression of the tight junction proteins occludin (**B**), claudin-4 (**C**), and claudin-2 (**D**) was assessed by Western blotting. For TEER measurements, data are displayed as log_2_-fold change, and for blots, as normalized arbitrary units measuring the expression ratio of the target protein and housekeeping protein β-actin. Results are shown as mean ± standard deviation of the mean (*n* = 6). Statistical significance was verified by one-way ANOVA followed by Tukey’s post hoc test. * *p* ≤ 0.05, ** *p* ≤ 0.01, *** *p* ≤ 0.001 in comparison to the untreated control group (first dataset) or the LPS-treated group (second and third datasets). # *p* ≤ 0.05, ## *p* ≤ 0.01 in comparison to the LPS-treated group (first dataset) or the Pio/FICZ-treated group (second and third datasets). “+” indicates the respective treatment at the left side of the graph was carried out; “-” indicates it was not. Dotted vertical lines separate datasets. The small horizontal line groups columns together. Lanes within a single blot were rearranged in order to align with the display of experimental groups; thin dotted vertical lines between bands indicate lanes that were spliced together. Original uncropped blots are shown in the Supplementary Materials. kDa = kilodaltons.

Knowing the pattern of secretion of the assessed cytokines into the extracellular milieu, we then assessed how such secretion could affect the integrity and permeability of the Caco-2 cells alongside THP-1 cells in the co-culture system. LPS treatment reduced transepithelial electrical resistance (TEER) values, indicating the epithelial monolayer became “leaky” due to contact with activated macrophages, and this effect was prevented by treatment with both FICZ and pioglitazone. While the beneficial effect elicited by pioglitazone was prevented by GW9662, indicating the effect is PPARγ-mediated, the effect elicited by FICZ was not prevented by CH223191, denoting the effect is not AhR-mediated, once again evidencing the breadth of anti-inflammatory effects exerted by FICZ that are not AhR-dependent. The effect on TEER exerted by pioglitazone was not inhibited by CH223191, which suggests no downstream modulation of AhR on PPARγ, but interestingly, the effects of FICZ were inhibited by GW9662, which leads to the conclusion that AhR-independent effects of FICZ on TEER values are mediated by PPARγ (Figure 6A).

Western blotting analysis of protein material harvested from the damaged epithelial layer revealed no changes in the expression of constitutive tight junction proteins occludin and claudin-4. In contrast, the expression of claudin-2, a pore-forming junctional protein that promotes gut permeability during IBD, was increased under inflammatory conditions, in accordance with decreased TEER values. Pioglitazone treatment had no effect in terms of preventing such an increase, suggesting its effect in preventing the LPS-mediated decrease in TEER values is likely linked to other junctional proteins. FICZ treatment, on the other hand, not only restored claudin-2 to non-inflamed levels but did so in a PPARγ-mediated manner and also, to a lesser extent, in an AhR-mediated manner, which in this case evidences the anti-inflammatory effect of FICZ relies on AhR and PPARγ functioning in tandem in a single pathway, in a manner aligned with the pattern seen for IL-6 secretion (Figure 6B–D).

## 3. Discussion

Development of AhR ligands for IBD therapy, albeit promising, has yet to bring new molecules into clinical use. The AhR is a very promiscuous receptor, capable of binding to many different molecules leading to pleiotropic effects, and it is paramount that its downstream mediators be identified in order to further advance the understanding of gut inflammation mechanisms it might regulate [11]. Likewise, other transcription factors that play a converging role amidst anti-inflammatory mechanisms have risen as likely candidates, not only as targets for drug development but also as biomarkers of disease progression and enablers of downstream attenuating effects. Among these, PPARγ has gained substantial attention in recent years as a key mediator of gut inflammation [34,45]. While it is known that both AhR and PPARγ regulate inflammation, especially involving macrophages, there are no studies attempting to link both factors establishing a mechanistic relationship, and even less so in IBD models. A small amount of evidence has demonstrated I3C, an AhR ligand found in diet, is capable of modulating PPARγ expression in colitis models in vivo and that thiazolidinedione derivates can, in turn, activate AhR, but these findings are products of serendipity rather than the result of focused studies [46,47].

Initial exploratory analysis using an RNA-seq dataset generated from gut macrophages obtained from UC subjects and healthy controls showed that levels of *AHR* and *PPARG* transcriptional activation are positively and significantly correlated in healthy individuals, but not in UC patients. Expression of *AHR* was also found to be increased, whereas expression of *PPARG* was decreased. Moreover, given that assessment of the dataset through PCA also showed both *AHR* and *PPARG* are the most important contributors in explaining model variance in healthy individuals, it is fair to assume the correlation between both factors seen in gut macrophages from healthy individuals likely represents a fine-tuned control between *AHR* and *PPARG*, where levels of one factor maintain the levels of the other in a homeostatic fashion. Indeed, mRNA as well as protein levels of PPARγ detected in gut biopsies have been described to be an indicator of disease progression and likely a good biomarker for diagnostics and of therapy success, especially in UC, where low levels are associated with active UC and higher levels indicate therapy-induced remission [48]. It has also been reported that decreased PPARγ levels in UC patients are accompanied by increased levels of IDO1, an upstream enzyme that metabolizes tryptophan into endogenous AhR ligands. This is in accordance with our findings, allowing us to speculate that an increase in AhR levels likely represent an attempt of the organism to restore downstream PPARγ signaling reinstating homeostasis, which is disrupted due to PPARγ levels being decreased during active disease [35,49].

A correlation between *PPARG* and *AHR* transcriptional activation being established after analysis of the RNA-seq dataset allowed us to move forward with further exploration of this relationship in vitro. In THP-1 macrophages, treatment with both AhR and PPARγ agonists failed to alter nuclear translocation of PPARγ. While PPARγ is mostly a nuclear protein and exerts its effects as a nuclear transcription factor canonically, it can be shuttled to the cytosol depending on ligand activity, which could point to additional protein–protein interactions rather than transcriptional mechanisms in response to PPARγ/AhR ligands. However, those interactions were not observed [50,51]. Still, treatment with pioglitazone decreased PPARγ expression, which allows us to infer there is a negative feedback loop mediating receptor expression based on PPARγ activity taking place in macrophages. Interestingly, FICZ also decreased PPARγ expression, suggesting it might also recruit and activate PPARγ leading to similar downstream events in the same vein as for pioglitazone treatment, which culminates in negative feedback regulation. A similar scenario where activation of PPARγ regulates its own expression has already been described in ovarian macrophages and in pancreatic β-cells [52,53]. As for AhR, pioglitazone did not induce any changes in either translocation or expression, while FICZ mediated AhR translocation from the cytoplasm into the nucleus as expected, as this is a known mechanism of FICZ leading to canonical AhR-mediated effects [54]. FICZ also decreased AhR expression, indicating a negative feedback loop also takes effect following activation with an AhR agonist, similar as seen for PPARγ. This effect, unlike for PPARγ, is known to occur through activation of ubiquitin/proteasome pathways, thus modulating protein levels rather than directly modulating protein expression, being an established cornerstone indicating AhR activation [55,56,57].

By further exploring how inhibitors affect FICZ-mediated AhR translocation into the nucleus, we found that PPARγ blockage with GW9662 also inhibited AhR translocation, meaning correct functioning of PPARγ activity is required for specific effects of AhR to be triggered, at least those relying on nuclear translocation; activation of ERα or binding to the ReIB/NF-κB complex, for example, which are known to take place after nuclear translocation, are likely to be hampered [54,58]. How exactly PPARγ controls AhR shuttling into the nucleus, however, requires further molecular transport studies; it is known that PPARγ is repressed by XAP2 and Hsp90 proteins, which are also responsible for sequestering AhR in the cytoplasm. Thus, inhibition of PPARγ might free up these proteins increasing the amount of non-translocated AhR by stabilizing it in the cytoplasm. Alternatively, direct inhibition of AhR through GW9662 could also be hypothesized [59,60,61].

Having determined the behavior of AhR and PPARγ under non-stress conditions in the face of either agonists or antagonists, we then proceeded to explore this relationship between both factors under inflammatory conditions by stimulating the THP-1 macrophages with LPS. Interestingly, we found that both FICZ and pioglitazone modulate the LPS-mediated expression of the costimulatory surface molecules CD80 and CD86 as well as the release of the cytokines TNF-α, IL-6, and IL-10. All of these effects could be inhibited by the blockage of pioglitazone-induced PPARγ activation. In contrast, after blocking FICZ-induced AhR activation, only CD86 and IL-6 were found to be affected, suggesting that, at least for the effects assessed in our study, pioglitazone relies mostly on PPARγ activation to exert its effects, while FICZ displays AhR-independent effects. As already mentioned, FICZ has been reported to display a plethora of effects regardless of binding to AhR, such as controlling TGF-β-induced collagen production in myofibroblasts and pMEK signaling in myeloblastic cells, so these effects are not unanticipated [62,63]. When examining the effects of pioglitazone and FICZ in combination with inhibitors, we primarily found that reciprocal inhibition was not observed: most pioglitazone-mediated effects were not blocked by CH223191, and similarly, most FICZ-mediated effects were not blocked by GW9662. This would initially suggest an absence of cross-talk between PPARγ and AhR in this model, if not for the effects seen for IL-6 secretion. Both pioglitazone-induced PPARγ activation and FICZ-induced AhR activation significantly suppressed IL-6 secretion, which was fully or partially restored by CH223191 and GW9662, respectively, providing evidence of interplay between both receptors, with IL-6 acting as the connecting factor. Certain flavones are reported to bind to AhR in macrophages, inhibiting NLRP3 inflammasome formation culminating in IL-6 inhibition, and this effect is described to rely on AhR translocation inhibited by CH223191, a phenomenon resembling the one here presented [64]. Considering that GW9662 also inhibited AhR translocation, meaning AhR translocation is a PPARγ-reliant process, the effect on IL-6 observed in our study might indeed rely on AhR translocating into the nucleus. Also, interferences with STAT1, STAT3, and NF-κB-mediated signaling in inflamed macrophages are known events downstream of both PPARγ and AhR activation, leading to reduced IL-6 secretion, meaning both receptors might share common downstream pathways [49,65,66,67]. It is important to note, however, and also attesting to the complexity of AhR signaling, that such observations are relegated to the macrophage inflammation model here employed, where in other non-inflammatory contexts, pathways leading to IL-6 secretion are induced by AhR rather than hampered. This is seen in adipocytes where kynurenine, another known endogenous AhR ligand, triggers STAT3/IL-6, leading to insulin resistance and in cancer cells where this pathway leads to tumor resistance [68,69].

Once cytokine release patterns by THP-1 macrophages were established, we assessed how these could influence epithelial permeability in a “leaky gut” co-culture model with Caco-2 cells. We assumed that pioglitazone and FICZ could likely prevent monolayer disruption as inflammatory cytokines were hampered by both ligands, which was indeed what we observed. Also, if these effects were to be disrupted by inhibitors of the other receptor, we hypothesized that this would be due to inhibition of IL-6 release, which we had identified as a central factor mediated by both receptors and known to be a major factor in disrupting the gut epithelial barrier in human IBD and in DSS colitis models [70,71]. Indeed, while CH223191 did not inhibit the effect of pioglitazone in restoring epithelial monolayer integrity, showing that the beneficial effect caused by pioglitazone relies on other soluble factors not necessarily linked to AhR modulation, GW9662 did abrogate the effects caused by FICZ. Perhaps unsurprisingly, FICZ-mediated effects on barrier integrity were not inhibited by CH223191, as FICZ is capable of exerting AhR-independent effects. However, this event in conjunction with the fact that GW9662 inhibited FICZ effects on barrier permeability demonstrates that PPARγ is pivotal for the actions of FICZ in this scenario, modulating both AhR-independent effects we have not yet identified and AhR-dependent inhibition of IL-6, which converge in restoring epithelial barrier integrity. A body of evidence supports these findings. It has been reported that the AhR ligand I3C is capable of increasing PPARγ expression in a TNBS-induced colitis model, leading to disease remission mediated by control of inflammation exerted by IL-22. We have also previously demonstrated that PPARγ activation is capable of activating ERK signaling, leading to control of inflammation at the endothelial barrier, an effect that is also mediated by FICZ but independent of AhR, as already evidenced by other authors. Furthermore, FICZ has been reported to induce activation of RXRα, a protein normally found dimerized with PPARγ and also crucial for its actions, in an AhR-independent manner during retinoic-acid-induced cell differentiation [46,62,72,73]. Moreover, LPS-treated THP-1 cells induced the expression of claudin-2 in Caco-2 cells, which was reversed by FICZ but not by pioglitazone. This effect of FICZ in turn was blocked by both GW9662 and, to a lesser extent, by CH223191. This is probably the most relevant finding, as this effect seen for claudin-2 aligns with the effects seen for IL-6; both are regulated by FICZ in an AhR- and PPARγ-dependent manner, which points to PPARγ being a downstream mediator of AhR activation. Increase in claudin-2 mediated by IL-6 is a known mechanism leading to epithelial disruption that is well-documented in animal models and in IBD patients, having been reported to be inhibited by AhR signaling [71,74,75,76]. Albeit several other studies have already shown the relevance of PPARγ for modulation of other junctional proteins, such as claudin-5 and ZO-1, and also for control of inflammatory cytokines especially in gut macrophages [77,78], to the best of our knowledge, PPARγ had not yet been described in scientific literature as being involved with the AhR-claudin-2 regulating pathway.

## 4. Materials and Methods

### 4.1. RNA-Seq Database Analysis

RNA-seq data were obtained from the Gene Expression Omnibus (GEO) database pertaining the study under accession number GSE123141, whose results were last updated in September 2021. This study is a prospective observational study that eventually obtained gut biopsies from IBD patients and from healthy donors. From the sample biopsies, gut macrophages were isolated, and their RNA was obtained, from which RNA-seq data were generated. Transcripts per million (TPM) values for *PPARG*, *AHR*, *CD80*, *CD86*, *IL6*, *IL10*, and *TNFA* genes out of data generated for a total of 8 healthy controls and 10 UC patients were used for further analysis. The dataset also comprises CD patients whose RNA-seq data we also analyzed, but results were not meaningful in any scenario by us tested, meaning the findings described in the present work are appliable mostly to UC patients rather than CD patients. Analyses pertaining to the CD group were thus not included in the present work. To our knowledge, this is the only RNA-seq dataset deposited in the GEO repository generated from intestinal macrophages of IBD patients. Additional information regarding subject gender, previous smoking history, and Mayo scores can be found in the Supplementary Material (Supplementary Table S1).

### 4.2. Cell Culture

Caco-2 cells (immortalized colon epithelial cells, ATCC no. HTB-37) and THP-1 cells (immortalized monocyte-like cells, ATCC no. TIB-202) were cultured in DMEM high-glucose medium (4.5 mg/mL) (Pan Biotech, Aidenbach, Germany) containing 10 mM HEPES (Pan Biotech) and 20% FBS (Merck, Darmstadt, Germany). Cells were kept at 37 °C under a controlled 5% CO_2_ atmosphere at sterile conditions. THP-1 cells were sub-cultured every 2–3 days by replacing culture medium by centrifugation. Caco-2 cells were sub-cultured every 2–3 days when about to reach 60% confluence by employing phosphate-buffered saline (PBS—0.15 M NaCl) (Carl Roth, Karlsruhe, Germany) containing 2 mM EDTA (Panreac AppliChem, Darmstadt, Germany). No cells were used after having reached passage 30.

### 4.3. THP-1 Differentiation

For differentiation of THP-1 cells from a monocytic phenotype into a macrophage phenotype, cells were seeded at a density of 2 × 10^5^ cells/well in 12-well plates in their usual culture medium alongside 10 nM phorbol myristate acetate (PMA) (Sigma Aldrich, St. Louis, MO, USA). Cells remained in culture under such conditions for 24 h. Afterwards, cells, now attached to the well bottom and spreading, were washed with PBS and given fresh PMA-free culture medium, being allowed to rest for further 24 h. At the end of this period, cells were washed with PBS and harvested with a 0.05% trypsin/0.02% EDTA solution (Pan Biotech) and checked for increased surface expression of CD11b and CD14 via flow cytometry to ensure they had acquired a macrophage phenotype (Supplementary Figure S2A,B). Macrophage-differentiated THP-1 cells were used for further co-culturing with Caco-2 cells.

### 4.4. Caco-2/THP-1 Co-Culture

Caco-2 cells were seeded in 12-well-sized (1.13 cm^2^ surface area) Transwell^®^ inserts (Greiner Bio-One, Frickenhausen, Germany) at a cell density of 5 × 10^4^ cells per insert utilizing the same culture medium as that used for maintenance cell culturing. Both apical and basolateral medium were replaced with fresh medium every 2–3 days. Cells were kept in such inserts until they formed a stable, confluent monolayer, which occurred at around day 14 after initial seeding, as verified by visual assessment by conventional light microscopy alongside the measurement of the transepithelial electrical resistance (TEER).

Simultaneously, on day 12 of Caco-2 culture in Transwell^®^ inserts, THP-1 cells were seeded in 12-well plates and PMA-differentiated for 48 h as described above. When both THP-1 cells had been differentiated into a macrophage phenotype and Caco-2 cells were fully confluent, inserts containing Caco-2 cells were transferred to the plate containing macrophage-differentiated THP-1 cells. Finally, the co-culture system was washed with PBS, and fresh culture medium containing test or control substances was added at different concentrations for further incubation for another 24 h.

### 4.5. Cell Treatments

Once the Caco-2/THP-1 Transwell^®^ co-culture system was established, in order to evoke agonistic/antagonistic responses, cells were treated with different combinations of pioglitazone (PPARγ-specific agonist) (Sigma Aldrich), FICZ (potent AhR agonist) (MeCDhemExpress, Monmouth Junction, NJ, USA), GW9662 (specific PPARγ antagonist) (Abcam, Cambridge, UK), and CH223191 (specific AhR antagonist) (Tocris, Bristol, UK), all at a final concentration of 10 µM; inhibitors GW9662 and CH223191 were also used at concentrations ranging from 0.5 µM to 10 µM in one specific experiment. Cells were also treated with 100 ng/mL lipopolysaccharide (LPS) from *E. coli* O26:B6 (Sigma Aldrich). All treatments were performed on the basolateral compartment of the co-culture system, right after inserts containing Caco-2 cells were placed atop of THP-1 cells. All treatments were carried out for 24 h. The final concentrations used were determined based on the highest concentration, which led to non-toxic effects following 7AAD viability tests, which themselves were tested based on concentrations used in previous studies of our group [36,73] (Supplementary Figure S2C).

### 4.6. Imaging Flow Cytometry

THP-1 cells were harvested after treatments with trypsin-EDTA (Pan Biotech), as previously described. After being washed with PBS, cells were then immediately fixed and permeabilized using a commercial kit designed for fixation and permeabilization of cells for the assessment of intracellular proteins, according to the manufacturer’s instructions (eBioscience, San Diego, CA, USA). Next, cells had unspecific epitopes blocked with 5% bovine serum albumin (BSA) (Sigma Aldrich) diluted in PBS for 30 min at room temperature (RT). Cells were washed with permeabilization buffer and incubated with primary antibodies for 30 min at RT. Afterwards, cells were washed again with permeabilization buffer for the removal of excess unbound primary antibodies and incubated with fluorochrome-conjugated secondary antibodies once more for 30 min at RT. Cells were also incubated with 1 µg/mL 4′,6-diamin-2-phenylindol (DAPI; Thermo Fisher, Waltham, MA, USA) at the same time as they were incubated with secondary antibodies. Lastly, cells were washed again with PBS and resuspended in fresh PBS. Stained cells were then imaged utilizing an Amnis^®^ Imagestream^®^X MKII device (Cytek Biosciences, Fremont, CA, USA), being excited by 405 nm and 488 nm lasers. At least 1000 events were acquired for each sample.

For the evaluation of single-cell images in order to assess nuclear translocation, cells were gated for best possible focus and then further gated for double-stained cells (nuclear staining and marker of interest). Around 40–60% of events were used for final analyses after setting gates. For each marker of interest alongside nuclear staining, corresponding cell images referring to the events at the end of the gating hierarchy were superimposed. Similarity indexes (SI) comparing the positioning of the superimposed images were generated utilizing IDEAS^®^ (v. 6.2) software (Cytek Biosciences). Cells were then grouped according to their SI values, namely, low being <0, medium being <1, and >0 and high being >1. The data here reported refer to SI values obtained for the high-similarity group. As this strategy is not usual for assessment of nuclear translocation of proteins, a detailed illustration of this gating strategy can be found in the Supplementary Material (Supplementary Figure S3).

For the assessment of the expression of a specific target, median fluorescence values (MFI) were obtained out of each sample and are reported here.

Details on primary antibodies and on fluorochrome-conjugated secondary antibodies used for imaging flow cytometry can be found in the Supplementary Material (Supplementary Table S2).

### 4.7. Conventional Flow Cytometry

THP-1 cells were harvested after treatments with trypsin-EDTA (Pan Biotech), much like for imaging flow cytometry. After being washed with PBS, cells right away had their unspecific epitopes blocked with 5% BSA (Sigma Aldrich) diluted in PBS for 30 min at RT. Afterwards, cells were again washed with PBS and incubated with fluorochrome-conjugated primary antibodies at previously standardized dilutions for a further 30 min at RT. Finally, cells were washed again with PBS in order for excess unbound antibodies to be removed and resuspended in PBS. Stained cells were then run through a CytoFLEX V5 flow cytometer (Beckman Coulter, Krefeld, Germany) and excited using 488 nm and 638 nm lasers. At least 10,000 events were acquired for each sample. MFI was used as a measure of the intensity of each staining.

For viability assays, cells were washed with PBS after harvesting and then immediately stained with 0.25 µg 7-aminoactinomycin-D (7-AAD) per 2 × 10^5^ cells (Beckman Coulter) for 30 min at RT. Cells were afterwards again washed with PBS and run through the same above-mentioned flow cytometry device. At least 10,000 events were acquired for each sample. The percentage of stained cells was used to indicate cell viability.

Details on fluorochrome-conjugated antibodies used for conventional flow cytometry can be found in Supplementary Material (Supplementary Table S2).

### 4.8. Enzyme-Linked Immunosorbent Assay (ELISA)

Cell supernatants from the basolateral and apical compartments of the co-culture system were harvested after treatments and centrifuged at 10,000 rpm for 10 min at 4 °C. The resulting clean supernatants were used for assessment of TNF-α, IL-6, and IL-10 by ELISA, according to manufacturer’s instructions (BD Biosciences, Heidelberg, Germany).

### 4.9. Transepithelial Electrical Resistance Measurements

TEER measurement was carried out in order to assess Caco-2 monolayer permeability as a reliable indicator of epithelial barrier integrity. Briefly, an epithelial volt-ohm meter (Millicell ERS-2) coupled to a “chopstick” electrode (Merck Millipore, Darmstadt, Germany) was properly disinfected and placed in culture medium used for maintenance of cells and allowed to equilibrate for 10 min. Afterwards, the electrode was inserted vertically into the Transwell^®^ system in a manner so that the longer electrode would be inserted into the basolateral compartment and the shorter electrode would be inserted into the apical compartment. The electrode was neither moved nor placed in contact with any surface of the Transwell^®^ system while measurements were being recorded. Measurements were taken as triplicates for each insert. An insert containing no cells and only culture medium was used as a blank. Average measurements from the blank (TEERb) were subtracted from measurements obtained from inserts containing cell monolayers (TEERv); this value was then multiplied by the insert surface area to generate final TEER values (TEERf), as follows: TEERf = (TEERv − TEERb) × 1.13 cm^2^. During Caco-2 differentiation, TEER measurements were taken every second day until cells became confluent and reached TEER values of at least 300 Ω. When Caco-2 cells were being treated, TEER measurements were taken at the moment compounds were added and then again 24 h later.

### 4.10. Western Blotting

After treatments, Caco-2 cells were harvested in radioimmunoprecipitation buffer (RIPA) (Carl Roth) containing protease/phosphatase inhibitor cocktails (Thermo Fisher) prepared according to the manufacturer’s instructions. Afterwards, lysates were briefly sonicated with the aid of an HD2070 ultrasonic homogenizer (Carl Roth) at light conditions (1 short pulse per second for 15 s at 30% of device’s power) and centrifuged at 11,000 rpm for 10 min at 4 °C. Supernatants were collected and had their protein content determined by bicinchoninic acid assay (BCA), according to manufacturer’s instructions (Thermo Fisher).

Protein homogenates containing 20 µg of protein were mixed with Laemmli reagent (Sigma Aldrich) and boiled at 95 °C for 5 min. The mixture was then loaded into 10% polyacrylamide gels (Thermo Fisher) and subjected to protein separation via sodium dodecyl-sulfate polyacrylamide gel electrophoresis (SDS-PAGE; Bio-Rad, Hercules, CA, USA) for 1 h at 150 V. Proteins were next transferred to polyvinylidene (PVDF) membranes (Thermo Fisher) utilizing a Power Blotter XL (Thermo Fisher) semi-dry transfer system for 1 h at 25 V. Membranes were afterwards washed with Tris-Buffer saline (Carl Roth) containing 0.05% Tween 20 (Carl Roth) (TBS-T) and blocked with a solution of TBS-T containing 5% powder milk for 1 h (Panreac Applichem). Right after blocking, membranes were incubated with primary antibodies overnight at 4 °C. Next, membranes were again washed with TBS-T and incubated with horseradish peroxidase (HRP)-conjugated antibodies for 1 h at RT. Membranes were then developed utilizing enhanced chemiluminescence reagents (ECL) (Thermo Fisher), and images were acquired utilizing a Celvin S chemiluminescence imaging device (Biostep, Burkhardtsdorf, Germany). Protein bands were quantified with ImageJ^®^ v. 1.54f [79], and their signal strength was assessed in relation to the signal obtained for β-actin, being reported as normalized optometric density units.

Details on primary and secondary antibodies used for Western blotting can be found in the Supplementary Material (Supplementary Table S2), as well as uncropped, full representative membranes (Supplementary Figure S4).

### 4.11. Statistics

All statistical analyses were performed using the software GraphPad Prism^®^ v. 6 (GraphPad Software, San Diego, CA, USA) and RStudio^®^ v. 4.4.1 (R Core Team, Vienna, Austria). Heatmap and PCA plots were built using the RStudio^®^ heatmaply package v. 1.5.0 [80] and the built-in princomp function, respectively. Datasets generated from the RNA-seq database were considered normal after passing D’Agostino–Pearson normality tests and were statistically analyzed with Student’s *t* and Pearson correlation coefficient parametric tests for the determination of differences and correlations between two groups, respectively. Experimental datasets were analyzed by one-way analysis of variance (ANOVA) parametric tests followed by Tukey’s post hoc test for comparison between groups. Results are shown as mean ± standard deviation of the mean. All experiments were repeated at least twice, with each repeat employing 2 to 3 biological replicates (*n* = min. 3, max. 6). *p* values were considered significant when ≤0.05.

## 5. Conclusions

**Figure 7:**
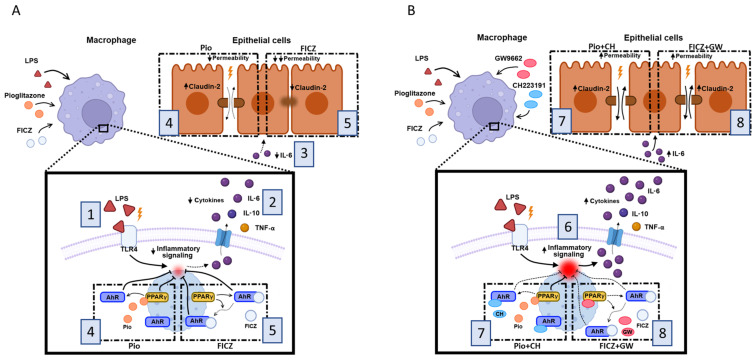
**Schematics summarizing the interplay between AhR, PPARγ, and ligands in the “leaky gut” Transwell^®^ co-culture model.** Under LPS-elicited inflammatory conditions, TLR4 activation in macrophage-differentiated THP-1 cells (**1**) leads to a series of downstream effects, namely, increased secretion of cytokines, such as TNF-α, IL-6, and IL-10 (**2**). These cytokines, especially IL-6, once released in the intercellular milieu, come in contact with the Caco-2 epithelial monolayer and disrupt its integrity by upregulating claudin-2 expression (**3**). In the presence of the PPARγ-agonist pioglitazone (Pio), all of these effects are attenuated with the exception of increased claudin-2 expression, which nonetheless culminate in reduced inflammatory status in THP-1 macrophages and preserved integrity of the Caco-2 monolayer (**4**) (left dashed squares). The AhR-agonist FICZ elicits similar effects while also restoring claudin-2 to basal levels (**5**) (right dashed squares) (**A**). In the presence of inhibitors, the anti-inflammatory response elicited by the agonists is mostly hampered, but in different ways (**6**). Blocking AhR with CH223191 (CH) prevents the effect of pioglitazone in attenuating IL-6 secretion by THP-1 macrophages, but as pioglitazone still elicits other PPARγ-mediated effects that do not rely on AhR, monolayer permeability is still restored, albeit not necessarily relying on claudin-2 (**7**) (left dashed squares). On the other hand, blocking PPARγ with GW9662 (GW) strongly inhibits the actions of FICZ, as it prevents both FICZ-mediated AhR nuclear translocation and also FICZ-mediated effects that do not rely on AhR translocation, which may or may not be AhR-mediated. This leads to increased secretion of IL-6 (and likely of other inflammatory mediators), culminating in increased LPS-mediated claudin-2 expression and subsequent increase in Caco-2 monolayer permeability (**8**) (right dashed squares) (**B**).

Overall, our findings deliver evidence for a cross-talk between AhR and PPARγ in preventing inflammatory effects exerted by LPS in the in vitro “leaky gut” Transwell^®^ co-culture model. The fact that effects of FICZ are prevented in most part by GW9662 supports our hypothesis that PPARγ is a downstream mediator of the anti-inflammatory effects of FICZ in both AhR-dependent and -independent manners (Figure 7). This could aid future research on development of AhR-based strategies for treatment of IBDs, either by facilitating development and repurposing of AhR agonists which might also target PPARγ, or by using PPARγ as a biomarker of therapeutic efficacy of AhR ligands.

## Data Availability

All data generated or analyzed during this study are included in this published article or are available as supporting material. The Caco-2/THP-1 Transwell^®^ co-culture model used in this study can be made available for basic as well as applied research and for preclinical drug development (GLP level).

## Supplementary Materials

The following supporting information can be downloaded at https://www.mdpi.com/article/10.3390/ijms252313072/s1.
