# Supplementary material for: AhR-Induced Anti-Inflammatory Effects on a Caco-2/THP-1 Co-Culture Model of Intestinal Inflammation Are Mediated by PPARγ
Source: Int J Mol Sci. 2024 Dec 5;25(23):13072. doi: 10.3390/ijms252313072 (PMC11642145; doi:10.3390/ijms252313072)
Supplement: Supplementary file 1 [file ijms-25-13072-s001.zip › ijms-3332989-supplementary.pdf]

Supplementary Table S1. Additional subject information from the RNA-seq dataset.

Subject Number	Patient ID	Disease	Gender	Smoking Status	Mayo Score	Immunosuppressive treatment
1	GSM3496520	Healthy control	M	Current smoker	N/A	N/A
2	GSM3496521	Healthy control	F	Previous smoker	N/A	N/A
3	GSM3496522	Healthy control	M	Never smoked	N/A	N/A
4	GSM3496523	Healthy control	M	Never smoked	N/A	N/A
5	GSM3496524	Healthy control	M	Never smoked	N/A	N/A
6	GSM3496525	Healthy control	M	Current smoker	N/A	N/A
7	GSM3496526	Healthy control	F	Previous smoker	N/A	N/A
8	GSM3496528	Healthy control	F	Never smoked	N/A	N/A
9	GSM3496529	Ulcerative Colitis	F	Never smoked	6	No
10	GSM3496530	Ulcerative Colitis	M	Never smoked	6	Yes
11	GSM3496531	Ulcerative Colitis	F	Previous smoker	2	No
12	GSM3496532	Ulcerative Colitis	M	Never smoked	8	Yes
13	GSM3496533	Ulcerative Colitis	F	Never smoked	6	No
14	GSM3496534	Ulcerative Colitis	M	Never smoked	6	No
15	GSM3496535	Ulcerative Colitis	M	Previous smoker	7	Yes
16	GSM3496536	Ulcerative Colitis	F	Previous smoker	9	No
17	GSM3496537	Ulcerative Colitis	M	Previous smoker	10	Yes
18	GSM3496538	Ulcerative Colitis	M	Never smoked	2	No

Information regarding subject ID, disease classification, gender, smoking status and Mayo score of UC patients are listed in the table above. Mayo score stands for "Disease Activity Index", and assesses disease severity based on daily stool frequency, rectal bleeding, mucosal appearance at endoscopy and physician's global assessment. All this information can be found under accession number GSE123141 at the open-access GEO database.

Supplementary Table S2. List of primary and secondary antibodies used.

1° antibodies				2° antibodies		
**Target**	**Dilution**	**Reference**	**Supplier**	**Dilution**	**Reference**	**Supplier**
**Imaging Flow Cytometry**						
PPARg	1/100	ab178860	Abcam	1/40	111-165-144	Jackson Immunoresearch
AhR	1/40	MA1514	Thermo Fischer	1/40	ab7002	Abcam
**Conventional Flow Cytometry**						
CD11b	1/100	333143	BD Biosciences	N/A	N/A	N/A
CD14	1/100	557742	BD Biosciences	N/A	N/A	N/A
CD80	1/10	PN IM 1976U	Beckman Coulter	N/A	N/A	N/A
CD86	1/10	555657	BD Biosciences	N/A	N/A	N/A
**Western Blotting**						

Occludin	1/1000	1404700	Thermo Fischer	1/5000	111-035-003	Jackson Immunoresearch
Claudin-2	1/1000	51-6100	Thermo Fischer	1/5000	111-035-003	Jackson Immunoresearch
Claudin-4	1/1000	LS-B8382	LSBio	1/5000	111-035-003	Jackson Immunoresearch
ß-actin	1/5000	PA1-16889	Thermo Fischer	1/5000	111-035-003	Jackson Immunoresearch

Antibodies used in all flow cytometry and western blotting experiments described in this work are listed in the table above alongside standardized dilutions, reference number and antibody supplier. Dilutions are optimal for flow cytometry when staining an amount of 2 x 10^5 cells in a final volume of 20 μL. Dilutions are optimal for Western Blotting when covering a whole membrane with an average volume of 5 mL. Secondary antibodies for conventional flow cytometry are not listed, as all primary antibodies used were already conjugated with a fluorochrome.

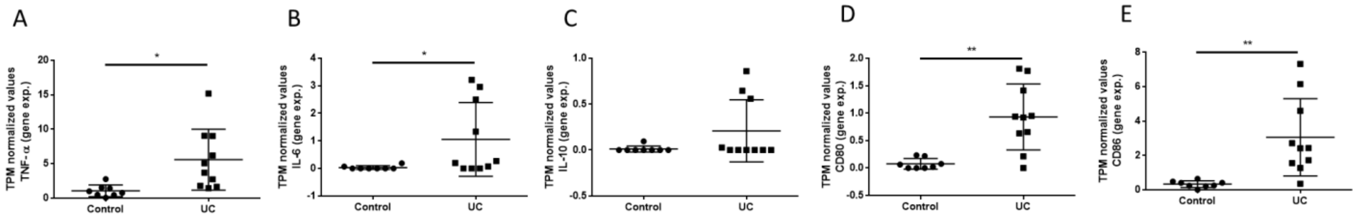

Supplementary Figure S1. Comparison of levels of macrophage inflammatory markers between healthy controls and UC patients in a GEO IBD dataset. RNA-seq data from an IBD dataset deposited at GEO was screened for expression levels of different macrophage inflammatory markers among healthy controls and UC patients. TPM values for each of the assessed parameters, namely TNF-α (A), IL-6 (B), IL-10 (C), CD80 (D) and CD86 (E), were individually plotted, averaged and compared between healthy controls and UC patients. Results are shown as mean ± standard deviation of the mean. Statistical significance was verified by Student's *t* test. * P ≤ 0.05, ** P ≤ 0.01 in comparison between groups. For all analyses, a total n = 8 for healthy controls and n = 10 for UC patients was utilized.

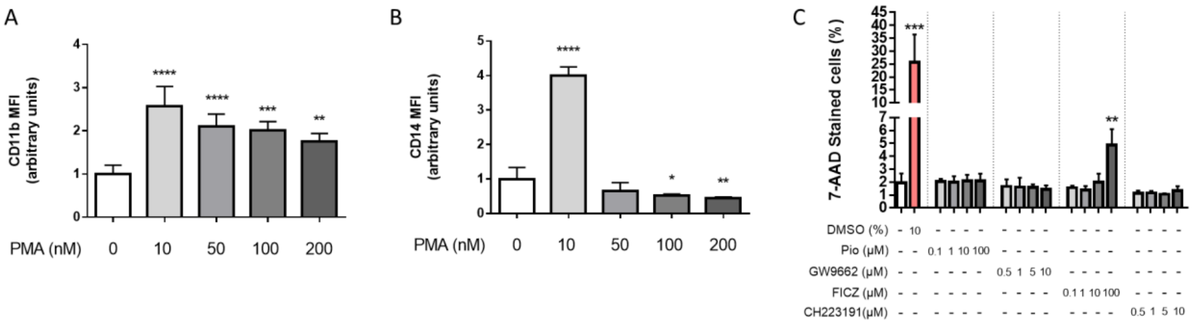

Supplementary Figure S2. THP-1 macrophage differentiation and viability testing. Monocytic THP-1 cells were treated with PMA at different concentrations for 24 h, washed, allowed to rest for further 24 h and then harvested. Surface expression of CD11b (**A**) and of CD14 (**B**) was assessed via conventional flow cytometry. Macrophage-differentiated THP-1 cells were treated with different concentrations of pioglitazone, GW9662, FICZ and CH223191 for 24 h. Cells were harvested after treatments and stained with 7AAD for assessment of cell viability; 10 % DMSO was used was positive control (**C**). Results are shown as mean ± standard deviation of the mean (n = 4). Statistical significance was verified by One-Way ANOVA followed by Tukey's post-hoc test. * P ≤ 0.05, ** P ≤ 0.01, ***P ≤ 0.001, **** P ≤ 0.0001 in comparison to the untreated control group. "+" indicates the respective treatment at the left side of the graph was carried out; "-" indicates it was not. Dotted vertical lines separate datasets.

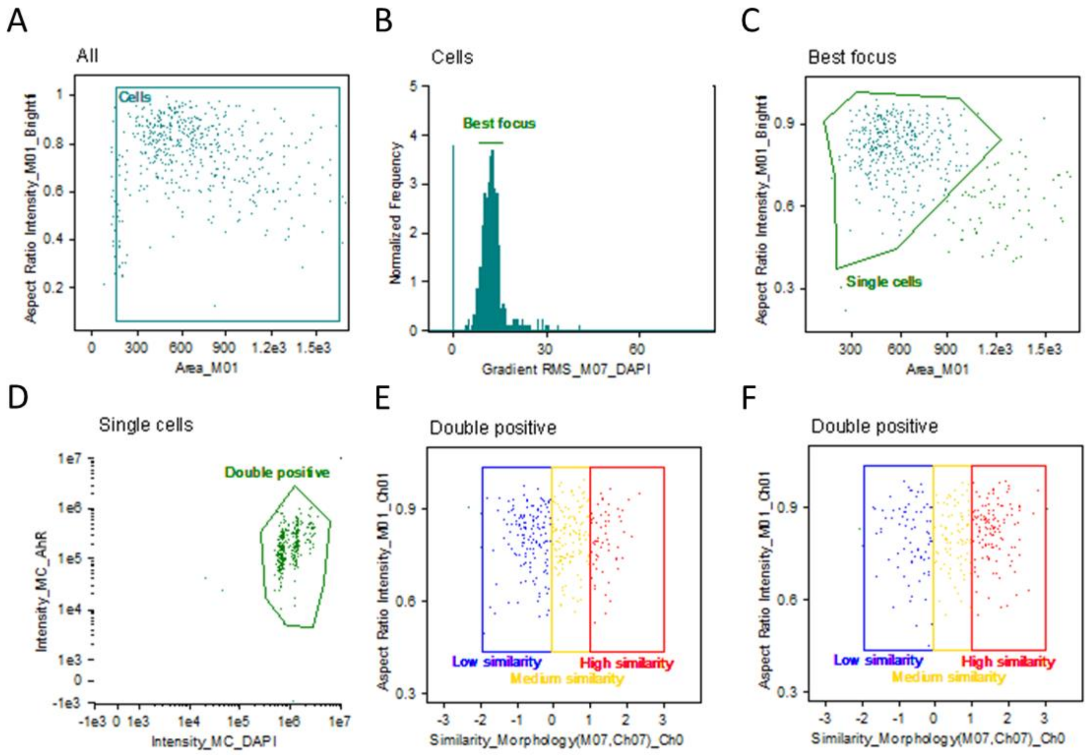

Supplementary Figure S3. Gating strategy for assessment of nuclear translocation. Cells were initially gated according to side and forward scatter, as per usual in flow cytometric analysis (**A**). Afterwards, the events registered at the best imaging resolution were used for further gating (**B**). By plotting signal height per signal area, events bellow a 45-degree threshold were excluded as being deemed doublets (**C**). Then, events displaying positive signal for both nuclear staining and marker of interest were used for determination of the similarity index (SI) (**D**). A final set of gates was drawn separating events of low, medium and high SI; for AhR translocation, a low number of events can be seen at the "high similarity" gate (highlighted in red) when referring to a control sample (**E**) and a higher number of events can be seen at this same gate when referring to a FICZ treated sample (**F**).

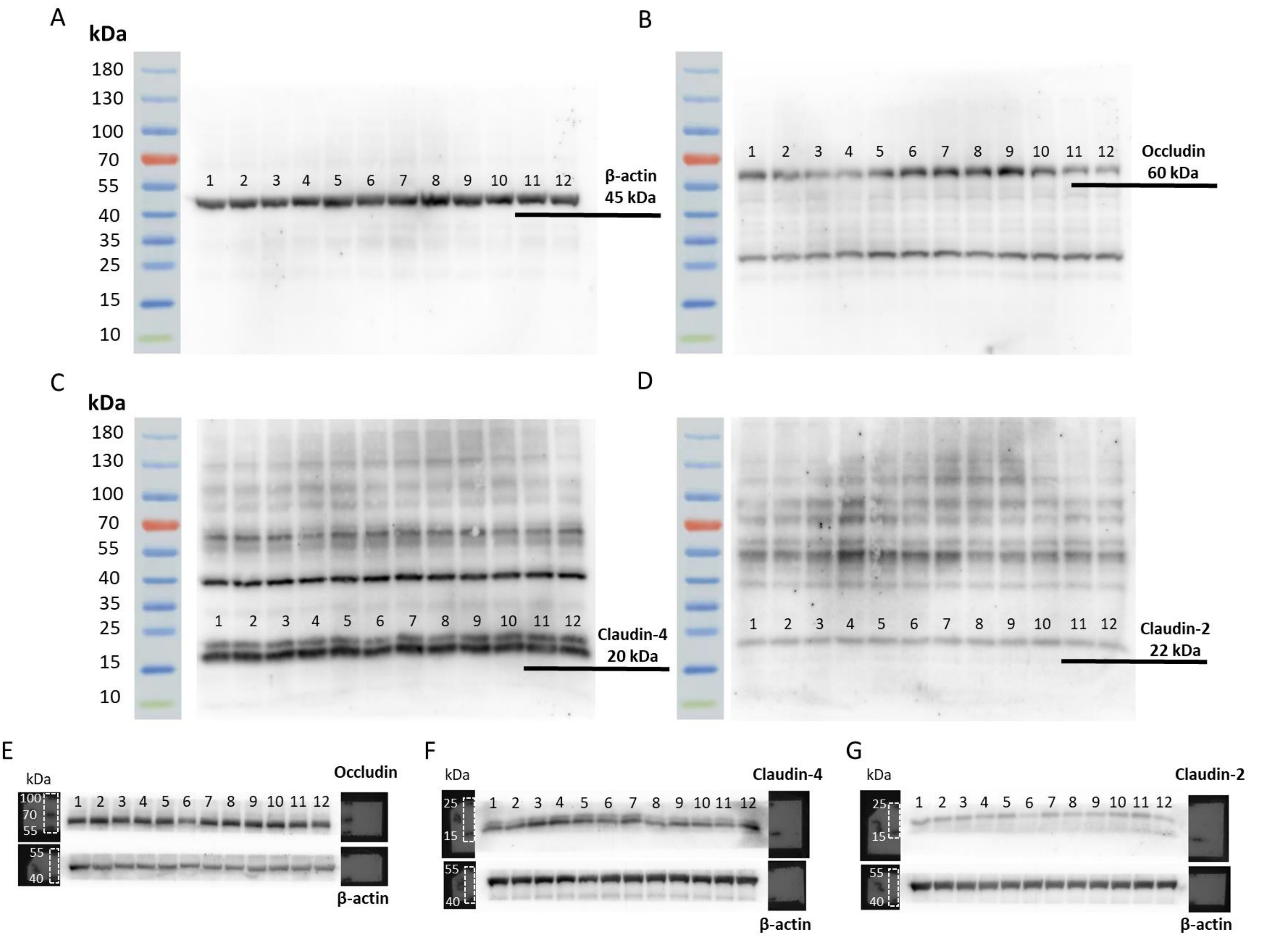

Supplementary Figure S4. Full uncropped Western Blotting membranes. Full uncropped Western Blotting membranes stained for each assessed target are shown alongside protein markers. The indicated bands in the first four figures show in a representative manner at which molecular weight the target proteins ß.actin (**A**), occludin (**B**), claudin-4 (**C**) and claudin-2 (**D**) were visualized. The next three figures refer to Figure 6 in the main text and represent the original blots which were spliced and rearranged for better visualization. In these figures, the upper row of bands represents Occludin (**E**), Claudin-4 (**F**) and Claudin-2 (**G**), while the lower row represents respective ß-actin bands in the same membrane; these refer to **B, C** and **D** in Figure 6, respectively. Dotted squared white lines in the left side of each figure indicate standard molecular weights in an unprocessed manner, which for reference can also be seen in the right side of each image. For all images, each lane represents a different experimental group: 1: control; 2: pioglitazone; 3: FICZ; 4: LPS; 5: LPS+pioglitazone; 6: LPS+FICZ; 7: LPS+GW9662; 8: LPS+CH223191; 9: LPS+pioglitazone+GW9662; 10: LPS+FICZ+GW9662; 11: LPS+pioglitazone+CH223191; 12: LPS+FICZ+CH223191. kDa = kilodaltons.
